# Monolayer organic thin films as particle-contamination–resistant coatings

**DOI:** 10.1038/s41598-023-37813-7

**Published:** 2023-07-14

**Authors:** Ruobin Jia, Brittany N. Hoffman, Alexei V. Kozlov, Stavros G. Demos, Alexander A. Shestopalov

**Affiliations:** 1 Laboratory for Laser Energetics, University of Rochester, Rochester, NY 14623-1299 USA; 2 Department of Chemical Engineering, University of Rochester, Rochester, NY 14627 USA

**Keywords:** Lasers, LEDs and light sources, Surface chemistry

## Abstract

Three organic monolayers coatings were developed and tested for their effectiveness to increase cleaning efficiency of attached microscale particles by air flows. The experiments were performed using silica substrates coated with these organic thin films and subsequently exposed to stainless-steel and silica microparticles as a model of contamination. Laser-induced–damage tests confirmed that the coatings do not affect the laser-induced–damage threshold values. The particle exposure results suggest that although the accumulation of particles is not significantly affected under the experimental conditions used in this work, the coated substrates exhibit significantly improved cleaning efficiency with a gas flow. A size-distribution analysis was conducted to study the adsorption and cleaning efficiency of particles of different sizes. It was observed that larger size (> 5-μm) particles can be removed from coated substrates with almost 100% efficiency. It was also determined that the coatings improve the cleaning efficiency of the smaller particles (≤ 5 μm) by 17% to 30% for the stainless steel metal and 19% to 38% for the silica particles.

## Introduction

The long-term performance of high-power laser systems can be adversely affected by particle contaminants that can be introduced into the system not only during the manufacturing of optical components, but also from the subsequent handling during optics installation and by particles introduced during the operation of the laser system^1^. Such particle contaminants can absorb or focus laser energy reducing the laser-induced damage threshold (LIDT) values and causing secondary contaminations from the damage sites that rapidly degrades the laser damage performance of the optical systems and thus, the laser power output^2–7^. In our recent study, we have investigated the properties of particles that accumulate near critical optics in the OMEGA EP grating compressor during periodic measurements over a 2-year duration. The study shows significant concentrations of micrometer- to nanometer scale particles inside the vacuum chamber. It is considered that the continuously generated particle load can introduce degradation of laser-induced damage performance and shorten optics’ lifetime^8^. With the continuous expansion of the utilization of high-power laser systems in various applications and operational environments, it is important to develop techniques and materials that can address this issue. Developing a solution can be a multifaceted problem that may involve understanding and mitigating the source of contamination, developing materials and optical component designs that can better handle the presence of contaminants but also develop technologies that can “resist” the accumulation of contaminants on high-value optics. For example, one can envision a process that enables the modification of the surfaces of optical devices with coatings that can reduce the adhesion of the particle contaminants^9–12^. Such coatings can help decrease the overall load of contamination and/or aid with the removal of the already accumulated particles using simple and practical methods, such as a gas-flow cleaning. However, it is important that such coatings do not alter the intrinsic LIDT values or the properties of the optical materials, and that they remain stable over time and during the system operation.

Organic monolayers covalently immobilized on the surfaces of inorganic materials can effectively modify their surface energy^13–15^. They are often used to prepare hydrophobic or hydrophilic interfaces to modulate the adhesion of various materials to inorganic substrates^16–18^. Such surface-energy modulation provides a robust way to control the strength of non-covalent Van der Waals interactions between the interfaces and various materials in the gas or liquid phase with overall areas of interaction ranging from angstroms to tens of micrometers. In particular, hydrophobic monolayers with low surface energy have been used to prepare interfaces with reduced absorptivity or self-cleaning properties^19–21^. A majority of the previous studies, however, have focused on the effectiveness of organic monolayers in cleaning with liquid solutions that are incompatible with large optical systems^22–24^. In addition, there are no past studies that examine the stability of such coatings in the operational environment of high-power laser systems, including the stability in a vacuum environment, exposure to laser pulses and their effect on the LIDT performance of the optical components.

This study details the performance of three monomolecular coatings developed to reduce and stabilize the surface energy on the surfaces of optical materials and increase the efficiency of a gas-flow cleaning in removing microscopic (~ 1–20 µm) particle contaminants. We examine the long-term stability of these coating in ambient air, vacuum, and under high-intensity laser radiation. We also examine the effect of these coatings on the LIDT performance of the underlying optical substrates. Finally, we measure the effectiveness of each coating in facilitating the removal of model metal and dielectric contamination particles with a simple nitrogen flow cleaning.

## Experiments and methods

### Materials

Fused-silica damage test optics (DTO) substrates (50-mm diameter, 7-mm thickness) fabricated in-house were utilized. The substrates were pre-cleaned by sonication in isopropanol at room temperature for 10 min. Subsequently the substrates were immersed in Nano-Strip (KMG Chemicals) to remove contaminations and create a fresh hydroxyl layer on the DTO interface (**Bare DTO**). Octyltrimethoxysilane (**M1**) and 1H, 1H, 2H, 2H-perfluorooctyltrimethoxysilane (**M3**) were purchased from Sigma-Aldrich and used as received. Carbene pre-cursor 2, 5-dioxopyrrolidin-1-yl 4-(3-(trifluoromethyl)-3H-diazirin-3-yl) benzoate (**M2**) was purchased from JR Medichem and used as received^25^. Other chemicals and solvents (isopropanol, toluene, dichloromethane, etc.) were purchased from Fischer Scientific at analytical grade or higher; isopropanol was filtered through a 0.2-μm filter before use. A blend of stainless-steel microspheres (1 to 22 μm, purity > 99.9%) or silica microspheres (1.86-μm and 9.2-μm diameter, monodispersed, purity > 99.9%) were obtained from Cospheric. Twenty mg of stainless steel particles were directly tested for each exposure, while 1 mg of silica particles of each diameter were blended for each particle exposure test.

### DTO functionalization

#### Methylated substrate (Me-DTO)

**Freshly prepared Bare DTO** substrate was immersed in a filtered (0.22-μm) toluene solution of **M1** (10 mM) and triethylamine (1 mM). The substrate was reacted with **M1** for 18 h at room temperature. The resulting **Me-DTO** substrate was rinsed with toluene and isopropanol and dried with a nitrogen flow.

#### NHS-terminated substrate (NHS-DTO)

Methylated **Me-DTO** substrate was reacted with **M2** in a custom-made vacuum chamber at 250 mTorr of pressure. As such, the **Me-DTO** substrate was positioned 10 cm away from the source containing 7.5 mg of **M2**. The source was heated to melt **M2** (~ 90 °C) for 5 min and the UV light (284 nm) was turned and the substrate was allowed to react with the **M2** vapors for another 90 min. After reaction, the substrate was rinsed with dichloromethane and isopropanol, and dried with the nitrogen flow.

#### Fluorinated substrate (F-DTO)

**Freshly prepared Bare DTO** was reacted with **M3** in a vapor phase in a glass vacuum jar containing 35 μL of **M3** and 3.5 μL of triethylamine. The jar was heated to 75 °C at 250 mTorr of pressure. The substrate was allowed to react with **M3** for 18 h. Subsequently, the functionalized **F-DTO** substrate was rinsed with isopropanol and dried with the nitrogen flow.

### XPS measurements

X-ray photoelectron spectroscopy (XPS) spectra were recorded using the Kratos Axis Ultra DLD XPS spectrometer equipped with a mono-Al x-ray source (1468.6 eV). The XPS spectra were collected using the widest lens and largest aperture analyzer settings (~ 600 × 900-μm substrate area). Multiple sweeps were recorded for the survey and regional scans (typically seven to ten sweeps) to increase signal-to-noise ratio. Unless specified, the electron collection angle Θ in all XPS measurements was zero. The XPS signal areas were measured using Casa XPS software.

### Contact-angle measurements

Surface contact angles were measured using VCA OPTIMA (AST Products, Inc.). Drops of water, ethylene glycol, and dimethyl sulfoxide (5 μL) at room temperature were deposited at a rate of 0.2 μL/s. Static, advancing, and receding angles were collected for each measurement.

### UV–Visible spectroscopy

The UV–visible spectra were recorded on Thermo Fisher EVO300 spectrometer. The compounds **M1**, **M2,** and **M3** were dissolved in carbon tetrachloride at 50 mM concentration. Absorption measurements were recorded from 190 to 1100 nm.

### Laser-induced–damage testing

Damage testing was performed using a laser system operating at 1053 nm with a tunable pulse duration between 0.6 ps and 100 ps. The system has been described in Ashe et al.^26^. Samples were tested in a vacuum environment (4 × 10^–7^ Torr) with *s*-polarized light at a 61° angle of incidence. Damage testing was performed in a single-shot (1-on-1) regime and damage was confirmed using Nomarski differential interference contract microscopy after testing.

### Ambient and vacuum stability tests

For an ambient stability test, the samples were flushed with dry nitrogen and sealed in polystyrene petri dishes with parafilm. For vacuum stability tests, the samples were placed inside a custom built stainless-steel vacuum chamber at 5 × 10^–6^ Torr. Samples were analyzed by XPS in every 30 days for a total test period of 90 days.

### Particle exposure tests

**Figure 1:**
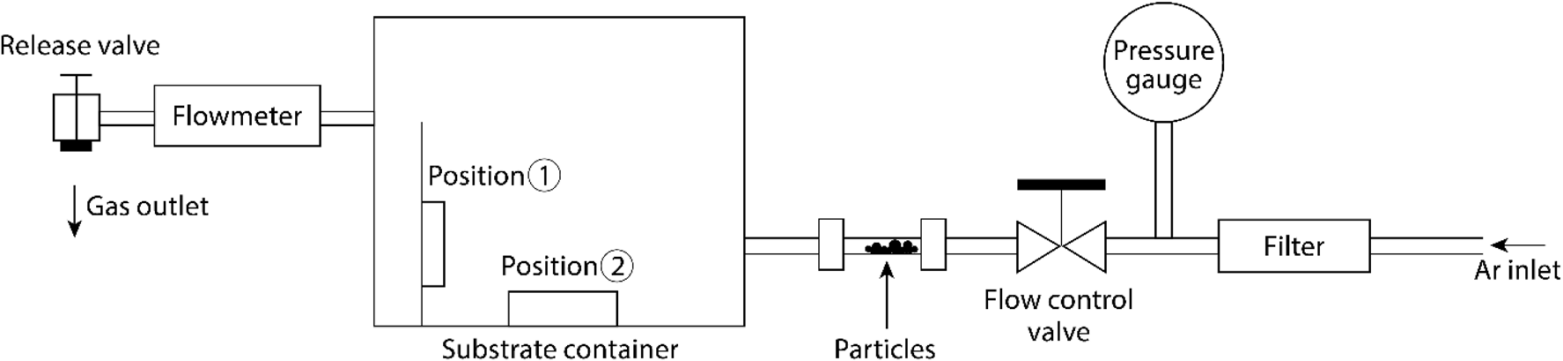
Schematic diagram of the particle exposure experimental setup.

**Bare DTO**, **Me-DTO**, **NHS-DTO**, and **F-DTO** were exposed to stainless-steel or silica particle mixtures to measure the differences in the particle attachment and the particle removal efficiency between the unfunctionalized substrates and the substrates modified with monomolecular films. The substrates were mounted in a sealed metal chamber and particle powders were carried into the chamber via an argon gas flow. The schematic representation of the particle exposure chamber is shown in Fig. 1. For each exposure, the sample chamber was pressurized to 2 bars with an argon gas. Next, the release valve was opened to allow the gas flow to transport the particles into chamber at a flow rate of ~ 1.4 L/s. Substrates were mounted on the chamber wall facing the gas inlet (position I) for metal particle tests and at the bottom of chamber (position II) for silica particle tests. Microscope images were collected on Zeiss Axio Imager A2m (DIC mode) to acquire particle density statistics for the sample substrates. Twenty images were taken on each particle-exposed substrate with an image size of 900 × 675 μm at 100 × magnification. The particle exposed substrates were subsequently cleaned with the dry nitrogen flow (~ 1-m/s velocity) for 6 s, and another group of 20 images was taken from each substrate to assess the particle removal efficiency. The images after the nitrogen cleaning were taken at nominaly the same coordinates as the images taken before the cleaning. The number of particles in microscope images was counted using ImageJ and Image-Pro software. To determine the standard deviation of the cleaning efficiency and to account for the non-uniform particle distribution over the sample area, the images before and after the cleaning were randomly combined into four groups (five images per group), and the standard deviation was calculated for the resulting four groups.

## Results and discussion

### Coating deposition and properties

**Figure 2:**
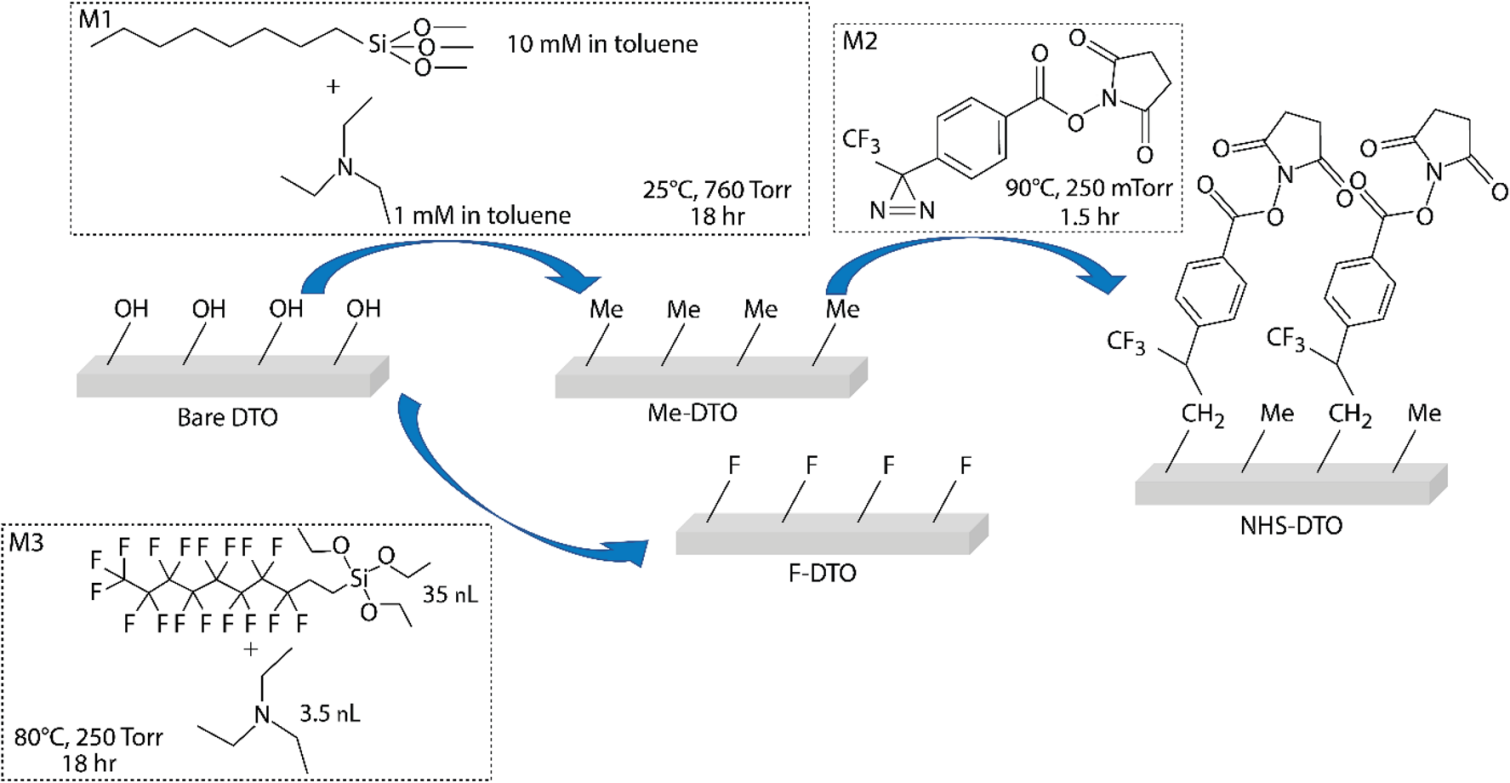
Functionalization steps of damage test optics (DTO’s) with silane- and carbene-based monolayers.

The coatings were deposited on fused-silica optical substrates (**bare DTO**) as discussed in the previous section. Silane molecules **M1** and **M2** were deposited on bare DTO from toluene solution (**M1**) and vapor phase (**M3**) to produce **Me-DTO** and **F-DTO** substrates. Diazirine molecule **M2** was attached to the **Me-DTO** substrate via a vapor-phase carbenylation reaction (**NHS-DTO**)^25^. The molecular structures and functionalization conditions of **Me-DTO**, **NHS-DTO** and **F-DTO** are shown in Fig. 2. **M1** and **M3** molecules are centrosymmetric alkoxy silanes that form self-assembled monolayer (SAM’s) on hydrophilic hydroxy-terminated surfaces via Si–O-Si bond formation. Both of these silanes are often used to reduce surface energy of oxide interfaces^27–30^. The **M2** molecule is a carbene precursor that can be used to modify inert methyl-terminated interfaces with stable and functional overlayers^25,31–34^. As such, it forms a stable overlayer via a carbene insertion into a C–H bond in mild, vapor-phase conditions. The terminal *N*-hydroxysuccinimide (NHS) group can be further modified to yield interfaces with desired chemical, physical, or biological properties^35,36^.

Following the reactions, the functionalized and unmodified DTO substrates were analyzed by XPS to determine the surface composition of the resulting interfaces. The atomic composition of unmodified and coated substrates is shown in Table 1. The **bare DTO** consists of silicon, oxygen, and carbon from the fused-silica material, which is confirmed by our XPS results; our cleaning protocol reduced the carbon content down to 3.8%, and no other impurities were found on the substrate within the sensitivity of our instrumentation. The **Me-DTO** consists of the same elements as bare substrate, with the detected carbon percentage increased to 11.7%, suggesting successful attachment of the **M1** silane to the DTO. XPS spectra of **NHS-DTO** contain F 1s signal and N 1s signal from the CF_3_ and NHS groups in **M2**, in addition to silicon, oxygen, and carbon signal. The **F-DTO** XPS spectra contain a strong fluorine signal from the fluorinated carbon chain in **M3**. In addition to the XPS analysis, we also confirmed the functionalization of the DTO substrates through water contact angle (WCA) measurements (Table 1). WCA measurements are commonly used to measure wettability and surface energy of the interfaces^37^. As such, clean DTO was completely wetted by water confirming its high surface energy and hydrophilicity.

**Table 1:** XPS element composition and water contact angles of bare and functionalized substrates.

	Elemental composition					
	C 1*s*	F 1*s*	N 1*s*	O 1*s*	Si 2*p*	Static, advancing, and receding WCA
Bare	3.8%	–	–	57.0%	39.2%	4°, wetting, wetting
Me-DTO	11.7%	–	–	51.8%	36.6%	92°, 109°, 88°
NHS-DTO	15.4%	2.0%	0.7%	45.6%	36.3%	73°, 86°, 62°
F-DTO	9.6%	11.4%	–	42.7%	36.3%	95°, 107°, 86°

DTO modified with alkylated (**Me-DTO**) and perfluorinated (**F-DTO**) SAM’s had significantly higher contact angles, confirming successful SAM formation. The contact angle of the **NHS-DTO** substrate was lower due to the presence of a more-polar NHS group. The WCA hysteresis—the difference between advancing and receding WCA – is in agreement with the previously published data for similar silane and carbene coatings^38–41^, indicating that the coatings were deposited as dense, uniform monolayers. Other than the homogeneity of coatings, the hysteresis is also affected by the roughness of substrates (measured as 0.37 nm for **bare DTO**). We also measured polar and dispersive components of the surface energies of the produced coatings using the Owens/Wendt method^42^. The polar and dispersive surface energies of the coatings were calculated from contact angle values provided by water, ethylene glycol, and dimethyl sulfoxide (Table 2), with calculation details provided in Table S1 and Fig. S1. The results indicate that **F-DTO** substrates have the lowest surface energy among the three coatings, and the polar surface energy of **NHS-DTO** is significantly higher than the two aliphatic silane coatings. These results suggest that **F-DTO** and **Me-DTO** should form weaker Van-der-Waals forces with polar and/or metallic contaminants, potentially aiding in their removal.

**Table 2:** Surface energies of the functionalized DTO substrates.

	Polar surface energy (mJ/m^2^)	Dispersive surface energy (mJ/m^2^)	Total surface energy (mJ/m^2^)
Me-DTO	0.4	42.1	42.5
NHS-DTO	11.0	24.2	35.2
F-DTO	0.8	32.9	33.7

### Optical and LIDT properties of coatings

**Figure 3:**
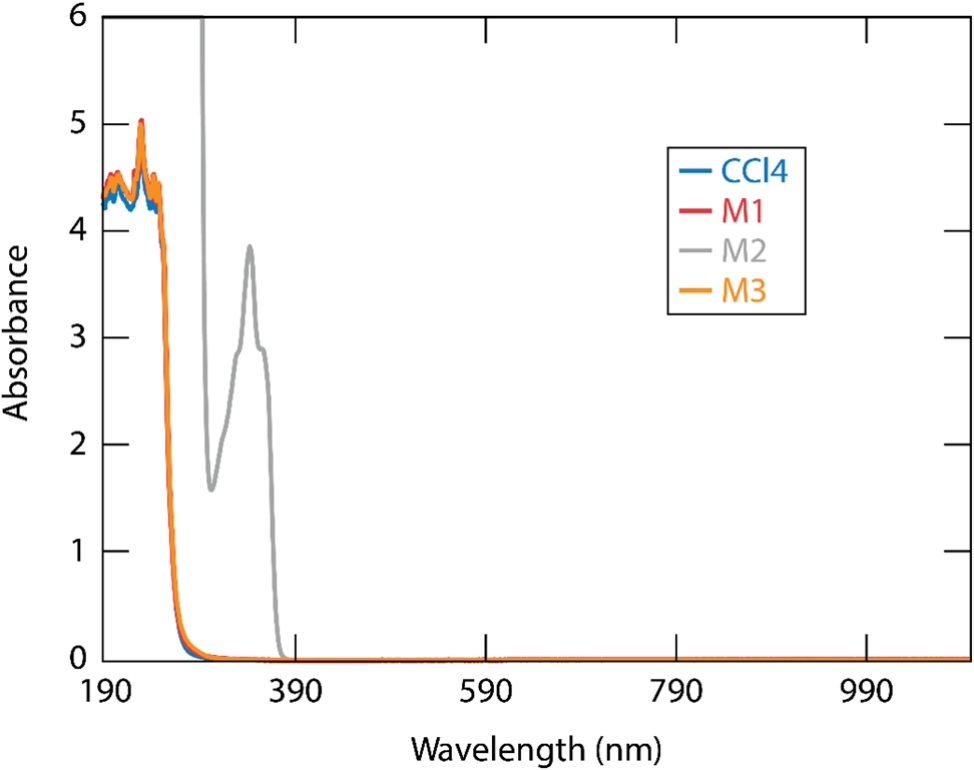
UV–Visible spectra of coating materials in carbon tetrachloride solution (50 mM); data reported for **M1**, **M2**, and **M3** with solvent CCl4 from 300 to 1100 nm.

To evaluate whether the coatings can be affected by exposure to the laser pulses, we measured the optical absorption spectra and the LIDT values of bare and coated DTO samples. The as-prepared coatings are less than 10 nm in thickness and their contribution to the absorption spectrum of the samples was not detectable using a conventional spectrophotometer; subsequently, the absorption measurements were performed using 50-mM solutions of **M1**, **M2**, and **M3** in CCl4. Specifically, for **M2**, the solution was set under white-light irradiation for 30 min so that the molecule could release the diazo group and reach a stable state. The absorption spectra in the 300-nm to 1100-nm spectral region are summarized in Fig. 3. We found that **M1** and **M3** do not show absorption within the measured wavelength range, whereas **M2** exhibits an absorption peak in the 300 to 380 nm spectra range, which corresponds to the aromatic ring structure in the molecule. No signal is observed for all compounds at wavelengths longer than 400 nm. Thus, we conclude that the coating materials do not exhibit linear absorption at the tested laser wavelength (1053 nm) but **M2** may exhibit absorption via three-photon excitation at high-peak-power intensities. Therefore, given that damage under ultrashort pulses at 1053 nm is driven by nonlinear absorption, the above results suggest that **M2** may exhibit a lower LIDT performance compared to **M1** and **M3**.

**Figure 4:**
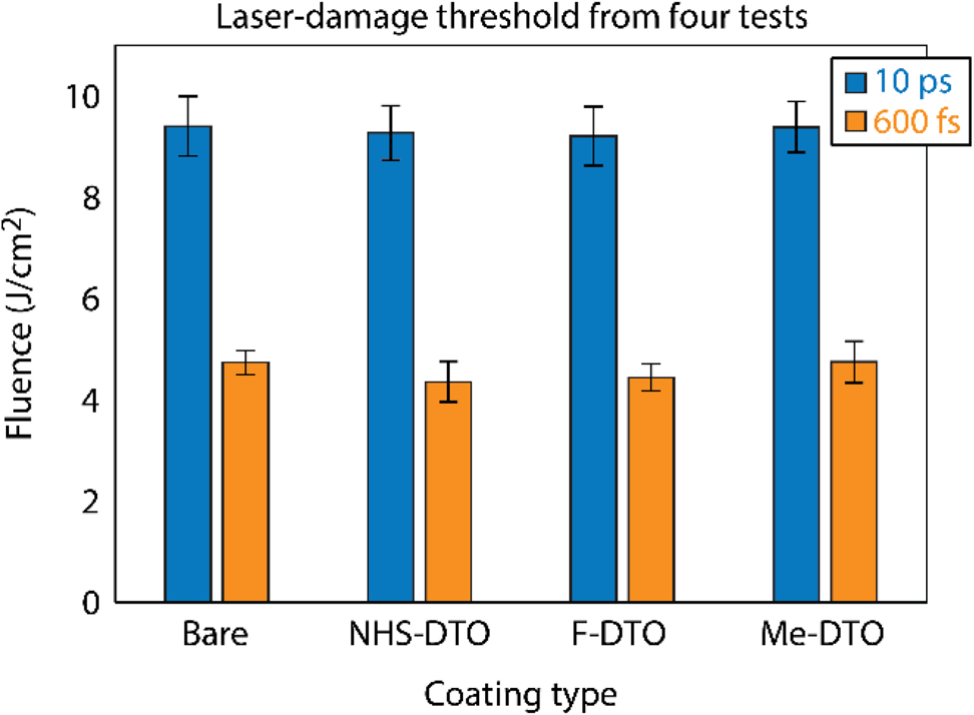
LIDT results from four test groups of coated and bare substrates under 10 ps and 600 fs pulse widths.

The interaction of the coatings with the laser pulses was further investigated by measuring the LIDT performance of the coated and bare DTO substrates. The damage-test experiments were performed using 10-ps and 600-fs laser pulses operating at 1053 nm. Each coated and bare DTO substrate was prepared and tested four times. Figure 4 shows the average LIDT values measured from four different samples of each coating type and the bare DTO. The LIDT values of the coated substrates are less than 1% different for the 10-ps pulses and less than 5% different for the 600-fs laser pulses when compared to the (unmodified) bare DTO. These results suggest that the LIDT of all coated substrates remained practically unaffected by the presence of the organic thin films investigated in this work. This includes coating **M2**, which the absorption spectra suggest will have a higher multiphoton absorption coefficient. This may be, in part, due to the very small thickness of the coatings and thus their ability to diffuse heating that is involved during the damage-initiation process^43^.

### Coating stability under exposure to laser pulses

To determine the effect of the laser exposure on the coating’s stability and density, we examined the elemental composition of the coatings following laser exposure. To explore the laser stability of the coatings, an area of each substrate was exposed to a 750-fs laser pulse at a fluence of 1 J/cm^2^. Table 3 shows the elemental composition of the coatings measured by recording XPS scans at and off the laser exposure sites. The results show that the elemental composition of all coatings was not significantly altered after the laser exposure. In these experiments, the laser-exposed areas were too small to accurately determine changes in the surface energies of the coatings. Considering the simple aliphatic nature and inert chemical reactivity of the **Me-DTO** and **F-DTO** coatings, however, we believe that the chemical structure of these monolayers were not significantly affected by the laser pulses.

**Table 3:** XPS interfacial element composition on and off laser exposure site.

	On exposure center					Off exposure site				
	C 1*s*	F 1*s*	N 1*s*	O 1*s*	Si 2*p*	C 1*s*	F 1*s*	N 1*s*	O 1*s*	Si 2*p*
Bare	10.9%	–	–	53.0%	36.1%	10.7%	–	–	52.6%	36.6%
Me-DTO	14.1%	–	–	49.9%	36.1%	14.3%	–	–	49.0%	36.8%
NHS-DTO	17.1%	1.9%	1.0%	45.5%	34.5%	17.0%	2.3%	0.7%	45.2%	34.8%
F-DTO	9.5%	8.3%	–	47.8%	34.4%	9.7%	6.1%	–	49.5%	34.6%

### Long-term coating stability in ambient and vacuum environments

**Figure 5:**
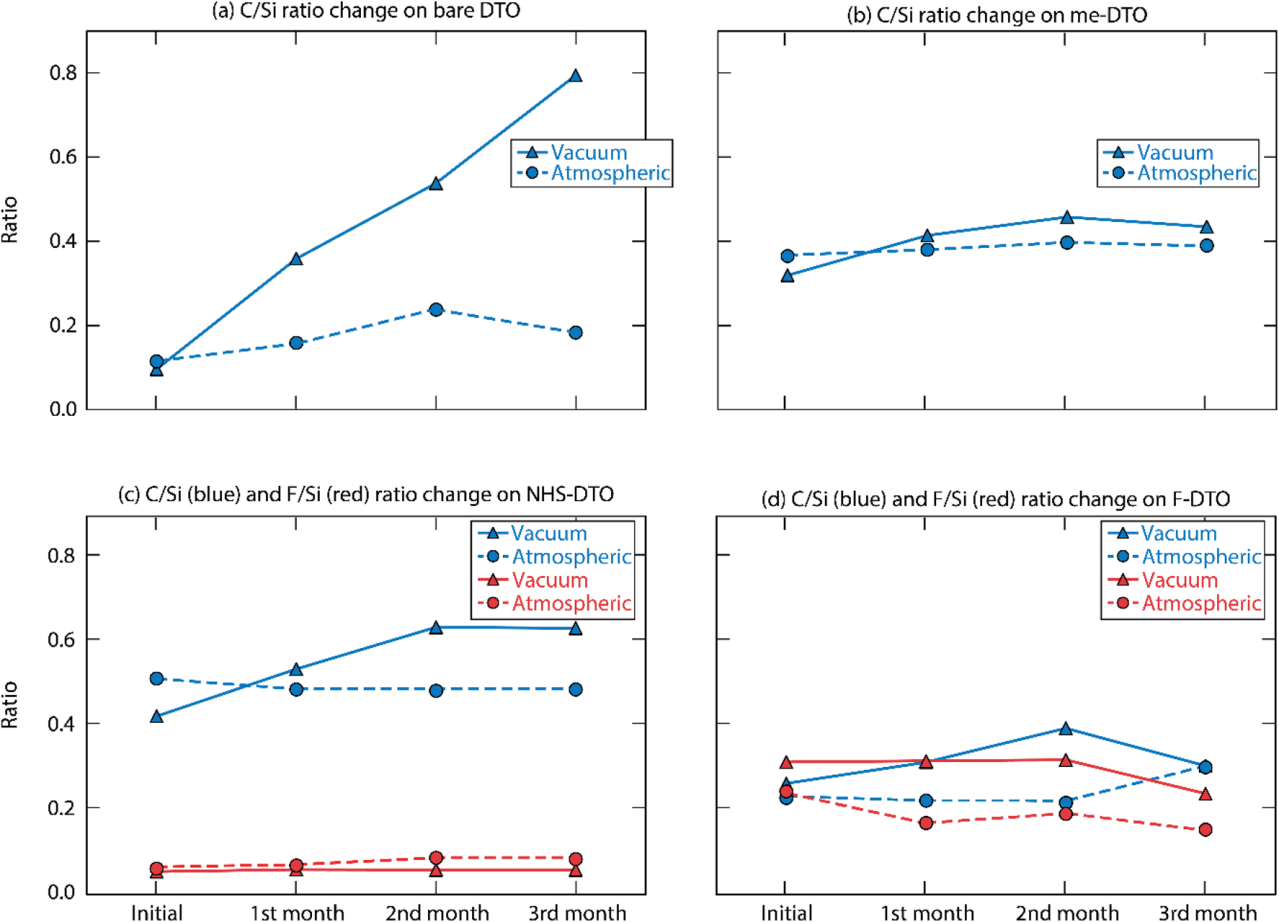
Stability of coatings presented by XPS peak area ratio. Namely C 1*s*/Si 2*p* ratio change in three months on (**a**) bare DTO and (**b**) **Me-DTO**; C 1*s*/Si 2*p* ratio and F 1*s*/Si 2*p* ratio change in three months on (**c**) **NHS-DTO** and (**d**) **F-DTO**.

Optical components that are most sensitive to particle contamination, such as optical elements in the pulse compressed section of ultrashort laser systems, operate under vacuum but are stored in an atmospheric environment. Thus, we tested the stability of the coated substrates in vacuum and atmospheric conditions. Two sets of samples were kept for 3 months under both vacuum and ambient conditions and analyzed for the composition changes every month. Specifically, the vacuum samples were placed in a stainless-steel vacuum chamber evacuated by a turbo pump to 5 × 10^−6^ Torr. The ambient samples were kept in plastic sample holders flashed with a dry nitrogen gas. The stability of the coatings was evaluated as atomic composition change by XPS. Stability results of the coatings are shown in Fig. 5a–d. We used C 1s signal as a characteristic element for the aliphatic **Me-DTO** coating (**M1** silane) and F 1s signals as a characteristic element for **F-DTO** and **NHS-DTO** samples (**M2** and **M3** molecules). The plots in Fig. 5 show the changes in C 1s intensity for **Me-DTO** and/or F 1s intensity for **F-DTO** and **NHS-DTO** as a function of the exposure time to vacuum or ambient environment. To normalize the data, the C 1s and F 1s area values were divided by Si 2p peak area from the substrate background. Figure 5a shows that the bare DTO accumulated a large amount of carbon content over time. In vacuum condition, the carbon composition increased throughout the testing period and reached to 830% of the initial value at the end of a three-month period. The bare substrate in atmospheric condition accumulated less carbon, reaching 208% of the initial value. Figure 5b–d show that all the coated substrates also adsorbed carbon inside the vacuum chamber, but with carbon composition increased only by 20% to 50% over the same three-month period. The coated samples exposed to the ambient conditions accumulated even less carbon. These results indicate that the coatings provide protection against nonspecific carbon accumulation in both vacuum and ambient environments compared to the bare DTO. Figure 5d shows that fluorine composition on **F-DTO** started to decrease after three months in vacuum and after one month in ambient, while the **NHS-DTO** exhibited the best elemental stability out of all coatings (Fig. 5c). Overall, the thin-film coatings investigated in this work demonstrated adequate stability both in vacuum and in ambient and reduce the amount of nonspecific carbon contaminates when compared to the bare DTO substrate.

### Particle adhesion analysis

Metal and glass particles are the most commonly found type of debris in optical system. The origin of these particles can vary between laser systems and can arise from laser damage on optical elements, stray beams impinging on optical holders and surrounding metal enclosures, or even generated at the target chamber where laser beam(s) are focused on specially designed targets to execute physics experiments. This particle-contamination mechanism is of particular concern in ultrashort laser systems, where the target chamber is directly connected with the pulse compression section of the laser system. Smaller particles (1–5 µm) have stronger interaction with the substrate interface and can be more difficult to remove. However, particles with submicrometer (subwavelength) size are not likely strongly affected by our 1053-nm laser but also far more difficult to study under the microscope^43^. Therefore, in the present work we selected model metal and silica particles with a size range of 1 to 20 μm for exposure tests.

#### Metal particle exposure

**Figure 6:**
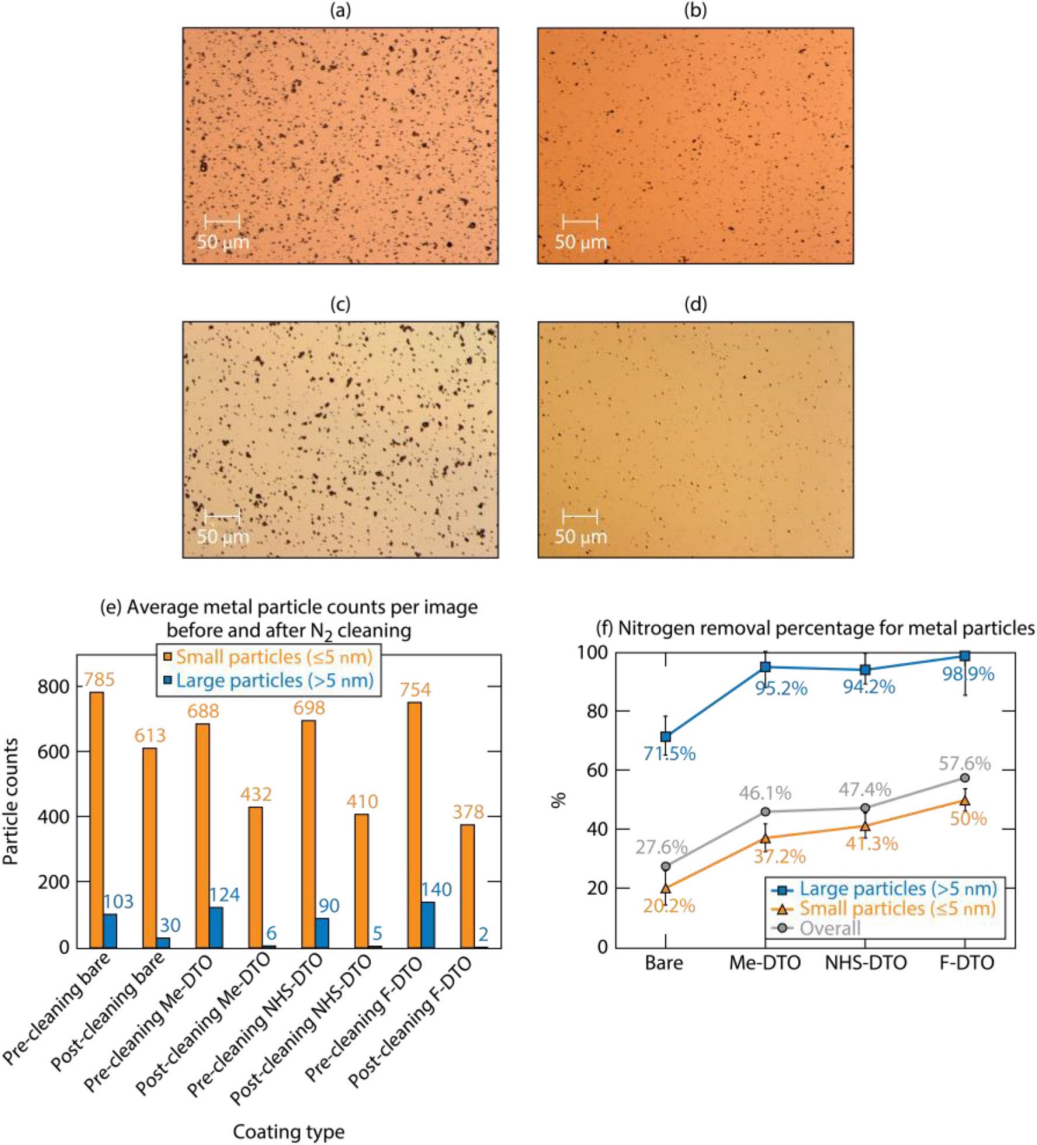
Sample metal particle exposure images (**a**) before and (**b**) after cleaning by nitrogen on bare DTO; sample metal particle exposure images (**c**) before and (**d**) after cleaning by nitrogen on **F-DTO**; (**e**) average metal particle counts per image and size distribution; (**f**) nitrogen flow removal efficiency for metal particles.

Stainless-steel particle powder having a distribution of particle diameters between 1 and 22 μm was used for exposure experiments. Twenty mg of particles were deposited onto the samples during each exposure process; 200 images were taken on pairs of coated substrates and corresponding bare DTO, and another 200 images were taken after the paired substrates were cleaned by nitrogen flow. Example images of stainless-steel particles dispersed on bare and **F-DTO** samples are shown in Fig. 6a–d before and after cleaning by nitrogen flow; the average particle counts per image is summarized in Fig. 6e. The data suggest that the number of attached particles is not significantly affected by the coatings. However, the particle counts on coated substrates after nitrogen cleaning is reduced by 32% to 41% compared to the particle reduction in bare substrates. To analyze the relationship between particle attachment and particle size, we analyzed this effect for the two particle size distributions as shown in the Fig. 6e. The results indicate that particles larger than 5 μm of size can be easily cleaned by gas flow with higher efficiency for the three coatings demonstrating a cleaning rate that approached 100%. To analyze the cleaning effect specifically, the nitrogen removal efficiency is shown in Fig. 6f. It can be seen that the coatings facilitated an increase of the removal rates of large (> 5 µm) and small (< 5 µm) particles by 24% to 27% and 17% to 30% respectively, compared to the bare sample. The results of metal particle exposure indicate that these coatings do not clearly affect the particle attachment during exposure, yet they do improve the cleaning efficiency of gas flow.

#### Silica particle exposure

**Figure 7:**
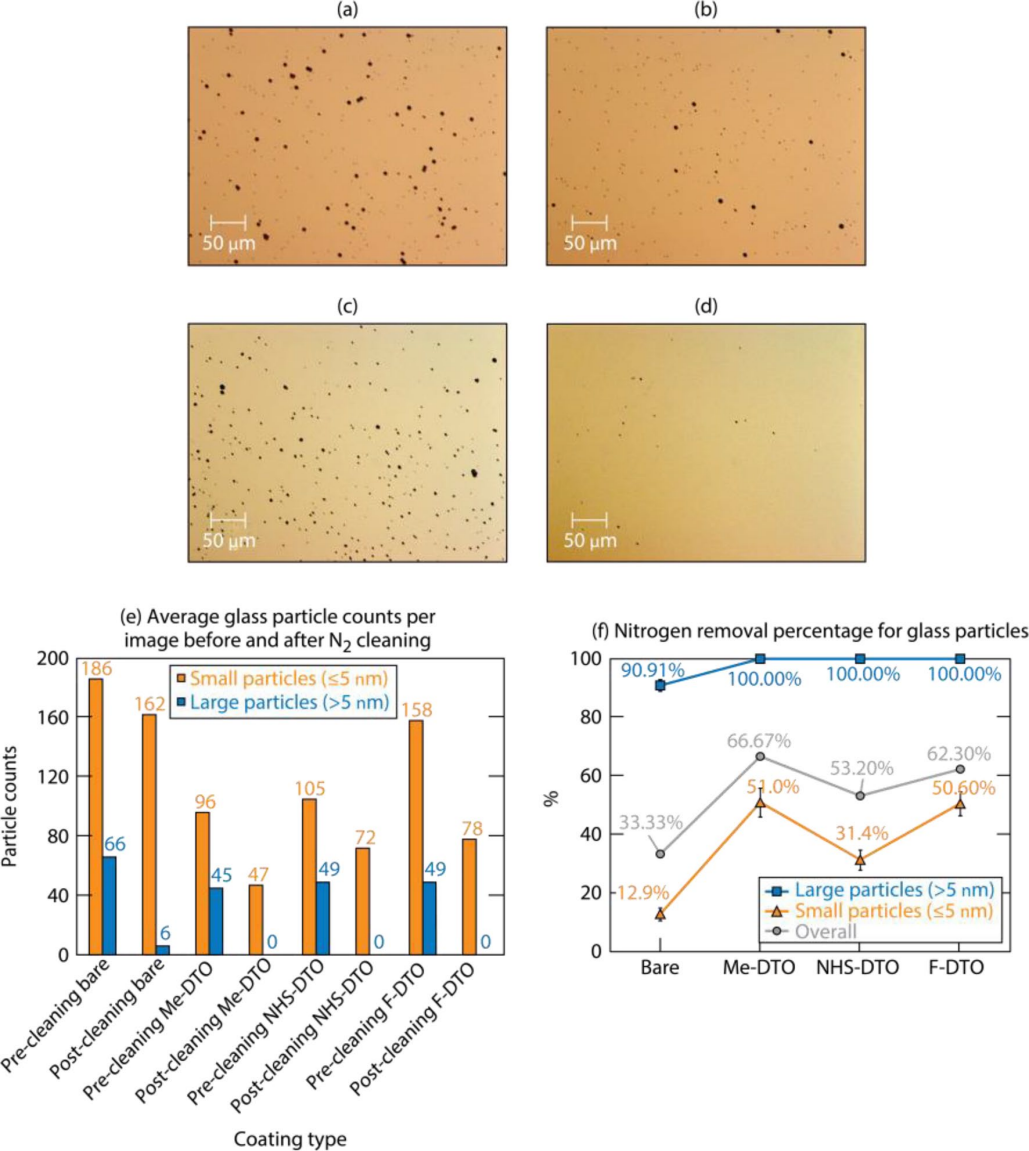
Sample silica particle exposure images (**a**) before and (**b**) after cleaning by nitrogen on bare DTO; sample silica particle exposure images (**c**) before and (**d**) after cleaning by nitrogen on **F-DTO**; (**e**) average silica particle counts per image and size distribution; (**f**) nitrogen flow removal efficiency for silica particles.

We conducted exposure tests of silica particles sized 1.86 μm and 9.2 μm in diameter. In each exposure process, the 1.86-μm particles (1 mg) and 9.2-μm particles (1 mg) were blended before loading into the system. To ensure that the number of silica particles attached to the substrates is consistent between the exposure tests, we modified the exposure condition by changing the mounting position of the substrates from vertical (position I) to lateral (position II) in the exposure chamber (Fig. 1). Twenty images were taken on each coated substrate along with bare DTO, and another 80 images were taken after the substrates were cleaned by nitrogen flow (Fig. 7a,b). Sample images of particle exposed areas on an F-DTO sample are shown in Fig. 7c and 7d. The results of particle counts and cleaning rate are shown in Figs. 7e and 7f. The data indicate that the coatings assist in improving the cleaning efficiency for silica particles. We observed that the adhesion of silica particles was reduced by all three tested coatings. The removal rates of large (> 5 µm) particles for all coated substrates reached approximately 100%, and the rates for small (< 5 µm) particles are improved by 19% to 38%. Similar to metal particles, the effect of coatings to reduce adhesion of silica particles can also be confirmed.

## Conclusions

We examined microparticle attachment effects on optics modified by organo-monolayer thin film coatings. Our results suggest that the coatings do not affect the damage threshold of the DTO and that they are stable for prolonged periods of time in vacuum and ambient environments. The particle exposure experiments with stainless steel and silica microspheres indicate that the coatings affect the strength of the noncovalent Van deer Waals interaction between the particle and the substrate, significantly reducing the lateral gas flow force needed to dislodge the particle from the interface. The relationship between the particle materials, the coating structures, and the amount of force required to remove them still remains to be studied. This study is, to the best of our knowledge, the first that examines the utilization of a thin film to act as a contamination mitigation tool in high power laser optical devices. The results demonstrate a significant improvement in the ability to remove the particle contaminants via gas cleaning and suggest that such a method may be a viable approach to address an important issue to increase the lifetime and operational envelope of optical components and reduce the cost of operation of such systems.

## Supplementary Information

Supplementary Information.

## Data Availability

Raw XPS data underlying the results presented in this paper are not publicly available at this time but may be obtained from the corresponding author (alexander.shestopalov@rochester.edu) upon request.
