# Supplementary material for: Monolayer organic thin films as particle-contamination–resistant coatings
Source: Sci Rep. 2023 Jul 14;13:11387. doi: 10.1038/s41598-023-37813-7 (PMC10349057; doi:10.1038/s41598-023-37813-7)
Supplement: Supplementary file 1 — Supplementary Information. [file 41598_2023_37813_MOESM1_ESM.docx]

Supporting Information

Monolayer Organic Thin Films as Particle-Contamination–Resistant Coatings

*Ruobin Jia^1, 2^, Brittany N. Hoffman^1^, Alexei V. Kozlov^1^, Stavros G. Demos^1^ and Alexander A. Shestopalov^1, 2^*

^1^Laboratory for Laser Energetics, University of Rochester, Rochester, New York, 14627, United States

^2^Department of Chemical Engineering, University of Rochester, Rochester, New York, 14627, United States

To calculate polar and dispersive surface energy components of the coated DTO substrates, we measured contact angles using water, ethylene glycol and dimethyl sulfoxide (**Table S1**).

**Table S1. Contact angle of the coated DTO substrates**

|  | Water Contact Angle | Contact Angle on Ethylene Glycol | Contact Angle on Dimethyl Sulfoxide |
| --- | --- | --- | --- |
| Me-DTO | 92° | 47° | 36° |
| NHS-DTO | 73° | 50° | 35° |
| F-DTO | 95° | 59° | 48° |

Subsequently, we used Owens/Wendt method to determine polar and non-polar components using linear fits (**Figure S1**).


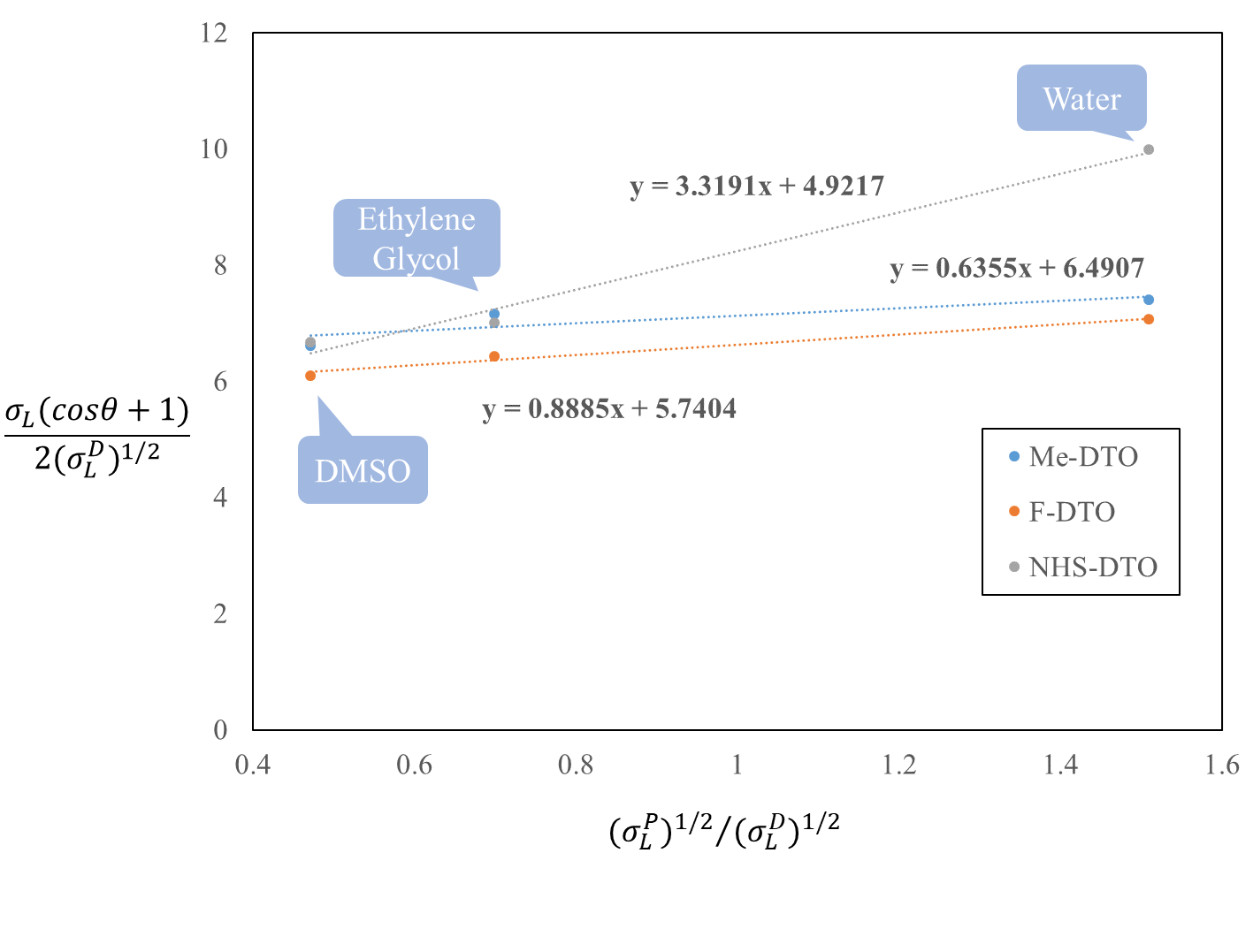


**Figure S1.** Owens/Wendt fit for coated DTO

Where $\sigma_{L}^{P}$,$\sigma_{L}^{D}$ and $\sigma_{L}$ are polar, dispersive and total surface tension of tested liquid, respectively; *θ* is the surface contact angle (rad).

The fitted functions are given in form of y=mx+b where m and b are defined as square roots of polar surface energy and dispersive surface energy of the surface (mJ/m^2^), respectively. The calculation results are reported in **Table 2** in the paper.
